# Characterization of γδT cells in lung of *Plasmodium yoelii-*infected C57BL/6 mice

**DOI:** 10.1186/s12936-021-03619-z

**Published:** 2021-02-15

**Authors:** Haixia Wei, Chenxi Jin, Anping Peng, Hongyan Xie, Shihao Xie, Yuanfa Feng, Anqi Xie, Jiajie Li, Chao Fang, Quan Yang, Huaina Qiu, Yanwei Qi, Zhinan Yin, Xinhua Wang, Jun Huang

**Affiliations:** 1 Key Laboratory of Immunology, State Key Laboratory of Respiratory Disease, Guangzhou Institute of Respiratory Health, The First Affliated Hospital of Guangzhou Medical University, Guangzhou, 510120 China; 2 Biological Resource Center, The Second Affiliated Hospital of Guangzhou University of Chinese Medicine, Guangzhou, 510120 China; 3 Zhuhai Precision Medical Center, Zhuhai People’s Hospital (Zhuhai Hospital Affiliated with Jinan University, Jinan University, Zhuhai, 519000 Guangdong China; 4 The Biomedical Translational Research Institute, Faculty of Medical Science, Jinan University, Guangzhou, 510632 Guangdong China

**Keywords:** *Plasmodium*, Lung, γδT cells, B cells, T cells

## Abstract

**Background:**

Malaria has high morbidity and mortality rates in some parts of tropical and subtropical countries. Besides respiratory and metabolic function, lung plays a role in immune system. γδT cells have multiple functions in producing cytokines and chemokines, regulating the immune response by interacting with other cells. It remains unclear about the role of γδT cells in the lung of mice infected by malaria parasites.

**Methods:**

Flow cytometry (FCM) was used to evaluate the frequency of γδT cells and the effects of γδT cells on the phenotype and function of B and T cells in *Plasmodium yoelii*-infected wild-type (WT) or γδTCR knockout (γδT KO) mice. Haematoxylin-eosin (HE) staining was used to observe the pathological changes in the lungs.

**Results:**

The percentage and absolute number of γδT cells in the lung increased after *Plasmodium* infection (*p* < 0.01). More γδT cells were expressing CD80, CD11b, or PD-1 post-infection (*p* < 0.05), while less γδT cells were expressing CD34, CD62L, and CD127 post-infection (*p* < 0.05). The percentages of IL-4^+^, IL-5^+^, IL-6^+^, IL-21^+^, IL-1α^+^, and IL-17^+^ γδT cells were increased (*p* < 0.05), but the percentage of IFN-γ-expressing γδT cells decreased (*p* < 0.05) post-infection. The pathological changes in the lungs of the infected γδT KO mice were not obvious compared with the infected WT mice. The proportion of CD3^+^ cells and absolute numbers of CD3^+^ cells, CD3^+^ CD4^+^ cells, CD3^+^ CD8^+^ cells decreased in γδT KO infected mice (*p* < 0.05). γδT KO infected mice exhibited no significant difference in the surface molecular expression of T cells compared with the WT infected mice (*p* > 0.05). While, the percentage of IFN-γ-expressing CD3^+^ and CD3^+^ CD8^+^ cells increased in γδT KO infected mice (*p* < 0.05). There was no significant difference in the absolute numbers of the total, CD69^+^, ICOS^+^, and CD80^+^ B cells between the WT infected and γδT KO infected mice (*p* > 0.05).

**Conclusions:**

The content, phenotype, and function of γδT cells in the lung of C57BL/6 mice were changed after *Plasmodium* infection. γδT cells contribute to T cell immune response in the progress of *Plasmodium* infection.

## Background

Malaria is a life-threatening disease caused by *Plasmodium* that are transmitted to people through the bites of infected female *Anopheles* mosquitoes. In 2019, there were an estimated 229 million cases of malaria worldwide. The estimated number of malaria deaths stood at 409,000 in 2019 [1]. Artemisinin has now become the world’s most effective drug for fighting malaria. Recently, there was a resurgence of malaria, partly as a result of increased resistance to artemisinin [2, 3]. To date, no vaccine has been shown to provide long-lasting benefits at a population level [4–7]. So, there is still a long way to go to achieve the goal of malaria elimination.

Besides respiratory and metabolic function, lung plays a role in immune system. It contains heterogeneous populations of innate and adaptive immune cells, such as T helper cells, macrophages, natural killer cells, gamma delta T cells (γδT cells), and others [8–10]. Malaria-associated acute lung injury (ALI)/acute respiratory distress syndrome (ARDS) is one of the main clinical complications of severe *Plasmodium* infection, which is one of the main causes of death [11–14]. However, the detailed mechanism of malaria-induced lung injury is unclear. Various immune cells are reported to participate in the process of malaria-associated ALI and ARDS in mice. For example, parasite-specific CD8^+^ T cells promote pulmonary vascular leakage and pulmonary edema [15, 16]. The B cells can protect the host from adverse lung pathological damage by secreting the IgA [17].

γδT cells represent a minor population of innate lymphocytes that can respond to the antigen without presentation [18]. γδT cells have multiple functions, producing different types of cytokines and chemokines, regulating the immune response by interacting with other cells [19]. The study of γδT cells in malaria was first published nearly 30 years ago [20], and recent findings showed that γδT cells play an important role in the protective immune response against *Plasmodium* [21]. Further evidence demonstrates that γδT cells are expanded in spleen, peripheral blood, lung, and liver of mice infected with different strains of *Plasmodium* [22–25]. γδT cells can regulate the anti-malaria immune response by interacting with other cells. For example, they can stimulate and recruit myeloid cells, promote the differentiation of CD4^+^ and CD8^+^ T cells by producing cytokines, like IFN-γ and TNF, and chemokines upon recognizing the soluble antigens released from parasites [22, 26–28]. There is an increasing body of evidence to support the fact that γδ T cells could modulate humoral immunity against *Plasmodium berghei* infection [29]. γδT cells were reported to involve in the pulmonary immunopathological injury caused by pathogenic organisms. For example, γδT cells could mediate influenza A (H1N1) induced lung injury by secreting interleukin-17A in mice [30]. γδT cells were found to mainly regulate the Th2 immune response in the lung of the mice infected with *Schistosoma japonicum* [31]. However, the potential roles of γδT cells during *Plasmodium* infection in the lungs C57BL/6 mice remains unclear. This research try to study the phenotype and function of γδT cells in the lung of C57BL/6 mice infected by *Plasmodium*, as well as the effects of γδT cells on T cells and B cells after *Plasmodium* infection.

## Methods

### Mice

Wild-type female C57BL/6 mice (6–8 weeks) were obtained from Animal Centre of Guangzhou University of Chinese Medicine (Guangzhou, China). γδT KO mice (B6.129P2-Tcrdtm1Mom/J, C57BL/6J genetic background) were acquired from JAX Stock (No. 002120). All protocols for animal use were approved to be appropriate and humane by the institutional animal care and use committee of Guangzhou Medical University (2012-11).

### Parasites and infection

The NSM strain of *Plasmodium yoelii* was purchased from the malaria research and reference reagent resource center (MR4). Frozen *P. yoelii* were thawed and maintained into C57BL/6 mice until the parasitaemia up to 10–15%. 6–8 weeks female C57BL/6 mice or γδT KO mice were infected with 1 × 10^6^ infected red blood cells (iRBCs) by intraperitoneal injection.

### Isolation of lymphocyte

Mice were euthanized at 11 days post-infection. Before obtaining the lung tissue, mice were perfused with sterile saline to remove the blood. The excised lung tissue was cut into small pieces and incubated in 5 ml of digestion buffer (collagenase IV/DNase I mix, Invitrogen Corporation) for 30 min at 37 °C. Digested lung tissue was pressed through a 200-gauge stainless-steel mesh and was then suspended in Hank’s balanced salt solution (HBSS). Lymphocytes were isolated using mouse lymphocyte separation medium (Dakewe Biotech) and density gradient centrifugation. The isolated cells were washed twice in HBSS and resuspended in complete RPMI 1640 medium supplemented with 10% heat-inactivated fetal bovine serum, 100 U/ml penicillin, 100 µg/ml streptomycin. After the lymphocytes were isolated, the cells were calculated by the blood cell counting plate with trypan blue staining.

### Antibodies

A detailed description of antibodies used in this study is provided in Table 1.

**Table 1:** Antibodies used in the study

Antibodies	Source	Identifier
Anti-mouse CD3 APC-cy7 (clone 145-2C11)	Biolegend	Cat. # 100330
Anti-mouse CD8 APC-cy7 (clone 54 − 6.7)	Biolegend	Cat. # 100714
Anti-mouse CD3 FITC (clone 145-2C11)	BD PharMingen	Cat. # 553062
Anti-mouse γδ TCR FITC (clone GL3)	BD PharMingen	Cat. # 553177
Anti-mouse CD19 Percp-cy5.5 (clone 6D6)	Biolegend	Cat. # 115534
Anti-mouse CD4 Percp-cy5.5 (clone GK1.5)	Biolegend	Cat. # 100434
Anti-mouse CD62L APC (clone MEL-14)	Biolegend	Cat. # 104411
Anti-mouse CD34 APC (clone MEC14.7)	Biolegend	Cat. # 119309
Anti-mouse CD11b PE-cy7 (clone M1/70)	Biolegend	Cat. # 101216
Anti-mouse PD-1 PE-cy7 (clone 29F.1A12)	Biolegend	Cat. # 135216
Anti-mouse CD80 PE (clone 16-10A1)	Biolegend	Cat. # 104708
Anti-mouse CD127 PE (clone ATR34)	Biolegend	Cat. # 135009
Anti-mouse PD-L1 Brilliant Violet 421 (clone 10F.6G2)	Biolegend	Cat. # 124315
Anti-mouse ICOS PE-cy7 (clone C398.4A)	Biolegend	Cat. # 3,153,520
Anti-mouse IFN-γ APC (clone XMG1.2)	BD PharMingen	Cat. # 554,413
Anti-mouse IL-17 APC (clone TC11-18H10.1)	BD PharMingen	Cat. # 506,916
Anti-mouse IL-21 APC (clone FFA21)	invitrogen	Cat. # 17-7211-82
Anti-mouse IL-5 APC (clone TRFK5)	Biolegend	Cat. # 504306
Anti-mouse IL-6 APC (clone MP5-20F3)	BD PharMingen	Cat. # 581367
Anti-mouse IL-4 PE (clone 11B11)	Biolegend	Cat. # 504104
Anti-mouse IL-17 PE (clone TC11-18H10)	BD PharMingen	Cat. # 559502
Anti-mouse IL-10 PE (clone JES5-16E3)	Biolegend	Cat. # 505008
Anti-mouse IL-2 PE (clone JES6-5H4)	Biolegend	Cat. # 503808
Anti-mouse IL-1α PE (clone ALF-161)	Biolegend	Cat. # 503203
Anti-mouse CD69 Brilliant Violet 421 (clone H12F3)	BD PharMingen	Cat. # 562920
Anti-mouse CD25 PE (clone BC96)	Biolegend	Cat. # 302606
TruStain FcX™ anti-mouse CD16/32 (Fc Block) (clone 93)	Biolegend	Cat. # 101320
APC Armenian Hamster IgG Isotype Ctrl Antibody (clone HTK888)	Biolegend	Cat. # 400911
Brilliant Violet 421 Armenian Hamster IgG Isotype Ctrl Antibody (clone HTK888)	Biolegend	Cat. # 400935
PE Armenian Hamster IgG Isotype Ctrl Antibody (clone HTK888)	Biolegend	Cat. # 400907
PE/Cy7 Armenian Hamster IgG Isotype Ctrl Antibody (clone HTK888)	Biolegend	Cat. # 400921

### Histology studies

Lungs were removed from mice and perfused three times with 0.01 M PBS (pH 7.4), fixed in 10% formalin, embedded in paraffin, and sectioned. The slice was stained by standard haematoxylin-eosin (HE) staining, and examined by light microscopy (Olympus ix71).

### Cell surface staining

Cells were washed twice with PBS and blocked in PBS buffer containing 1% BSA for 30 min. Cells were then stained with specific antibodies for the cell surface antigens for 30 min at 4 °C in the dark. The phenotypes (1 × 10^6^ cells per run) were analysed using flow cytometry (Beckman CytoFLEX) and CytExpert 1.1 (Beckman Coulter Inc.). The single nuclear cells were gated to exclude the dead cells and doublet. For gating CD3^+^ γδTCR^+^ cells, CD3^+^, CD3^+^ CD4^+^, CD3^+^ CD8^+^, CD3^−^ CD19^+^ cells, fluorescence minus one (FMO) controls were used. For other surface makers, isotype controls were used. 1,000,000 cells were used for cell surface staining, and 300,000 events were collected for each tube.

### Cell intracellular cytokine staining

1.5 × 10^6^ cells were resuspended in complete RPMI 1640 medium, then stimulated with phorbol 12-myristate 13-acetate (PMA) (20 ng/ml, Sigma) and ionomycin (1 µg/ml, Sigma) for 1 h. Brefeldin A (BFA, 10 µg/ml, Sigma) was added and incubated for 4 h. Cells were washed twice in PBS and stained with specific antibodies for the cell surface antigens for 30 min at 4 °C in the dark. Cells were fixed with Fixation and Permeabilization Solution (BD Biosciences) for 20 min at 4 °C in the dark. Next, cells were stained with specific antibodies for each cytokine. The results were analysed using flow cytometry (Beckman CytoFLEX) and CytExpert 1.1 (Beckman Coulter Inc.). The single nuclear cells were gated to exclude the dead cells and doublet. For gating CD3^+^ γδTCR^+^ cells, CD3^+^, CD3^+^ CD4^+^, CD3^+^ CD8^+^, CD3^−^ CD19^+^ cells, FMO controls were used. Isotype controls were used for intracellular cytokines staining. 1,500,000 cells were used for cell intracellular cytokine staining, and 300,000 events were collected for each tube.

### Statistics

The differences between the two groups were analysed in Prism (GraphPad Software) using a two-tailed Student’s t-test with equal variance and normal distributions. To compare more than two groups, one-way ANOVA and LSD test by SPSS software package were used with equal variance and normal distributions. Mann-Whitney U test was used with unequal variance or abnormal distributions. The statistical significance was defined as *p* < 0.05.

## Results

### *Plasmodium* infection induces the accumulation of γδT cells to the lung

**Fig. 1:**
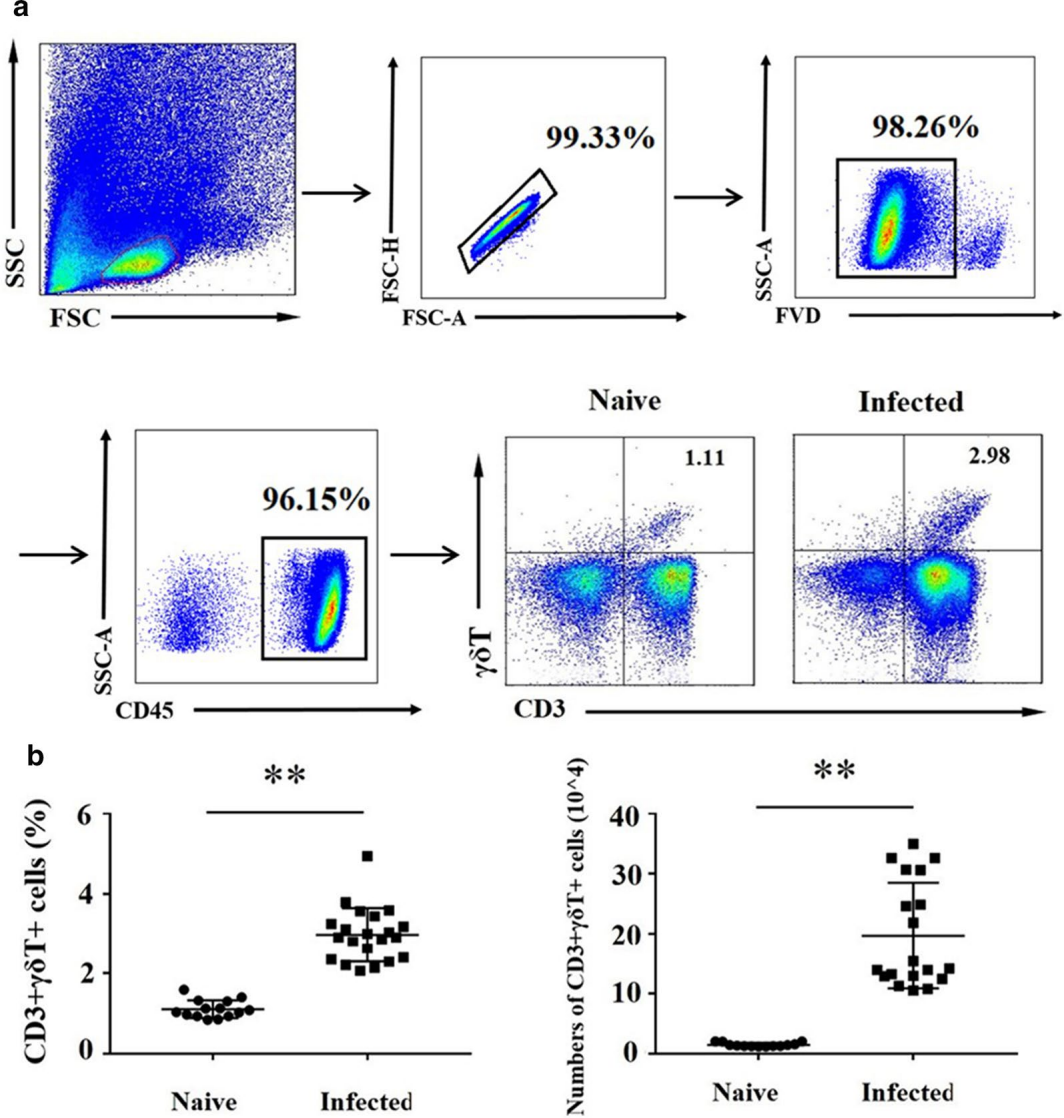
*Plasmodium yoelii* infection induces the accumulation of γδT cells to the lung. Pulmonary lymphocytes were stained with anti-CD3 and anti-γδTCR fluorescent mAbs. The expression of CD3 and γδT on lung lymphocytes of naive and infected mice were analysed by FCM. FMO controls for CD3 and γδTCR were included in the staining protocol. **a** One representative sample. All the doublet cells, dead cells, and non-lymphoid cells were excluded in this study. **b** Comparison of the percentage and absolute number of CD3^+^ γδT^+^ cells from the naive and infected groups. 5–7 samples were prepared for each group, and the experiments were repeated three times. **p < 0.01

To evaluate the change of γδT cells in the lung of the *Plasmodium*-infected mice, C57BL/6 mice were euthanized, and the lungs were removed 11 days post-*Plasmodium* infection. Single cell suspensions were prepared and calculated by the blood cell counting plate with trypan blue staining. The percentage and the absolute number of CD3^+^ γδTCR^+^ cells were determined by FCM (Fig. 1a). The staining strategy is shown in Additional file 1: Table S1. All the doublet cells, dead cells, and non-lymphoid cells were excluded in this study. As shown in Fig. 1b, the percentage of CD3^+^ γδTCR^+^ cells in the infected group was significantly higher than that in the naive group (naive: 1.11 ± 0.06 %, infected: 2.98 ± 0.15%, *p* < 0.01); and the absolute number of CD3^+^ γδTCR^+^ cells post-infection was also significantly increased (naive: 1.46 ± 0.09 per 10^4^ cells, infected: 19.7 ± 2.03 per 10^4^ cells, *p* < 0.01).

### Surface maker changes and cytokines released in the pulmonary γδT cells

**Fig. 2:**
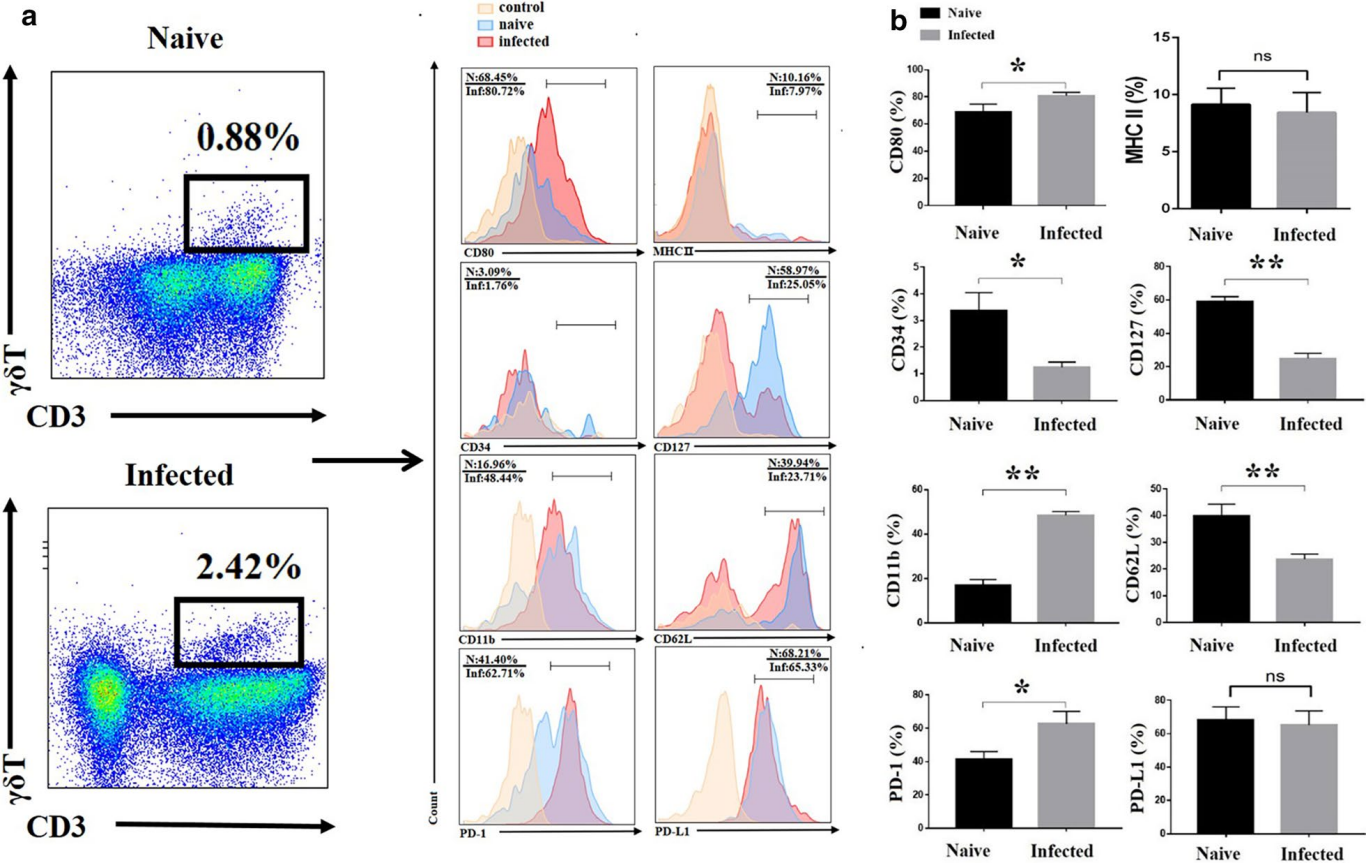
The expression of surface molecules on γδT cells. Pulmonary cells were stained with fluorescent mAbs against mice: CD3, γδTCR, CD80, CD34, CD127, CD62L, CD11b, PD-L1, and PD-1. FMO controls for CD3, γδTCR and isotype controls for CD80, CD34, CD127, CD62L, CD11b, PD-L1, and PD-1 were included in the staining protocol. **a** One representative FCM analysis. **b** Comparison of the expression of different surface molecules on CD3^+^ γδTCR^+^ cells from the naive and infected groups. 5–7 samples were prepared for each group, and the experiments were repeated three times. *p < 0.05, **p < 0.01

To study the phenotypic changes of γδT cells post-infection, single pulmonary cells from the naive and infected mice were stained with the different surface markers labelled with fluorescence: CD80, MHC II, CD34, CD127, CD62L, CD11b, PD-L1, and PD-1. The staining strategy is shown in Additional file 1: Table S2. As shown in Fig. 2, more CD3^+^ γδT^+^ cells were expressing CD80, CD11b, and PD-1 post-infection (*p* < 0.05), while less CD3^+^ γδT^+^ cells were expressing CD34, CD127, and CD62L in the infected mice (*p* < 0.05). There was no significant difference in the percentages of MHC II^+^ and PD-L1^+^ γδT cells between the naive mice and infected mice (*p* > 0.05).

**Fig. 3:**
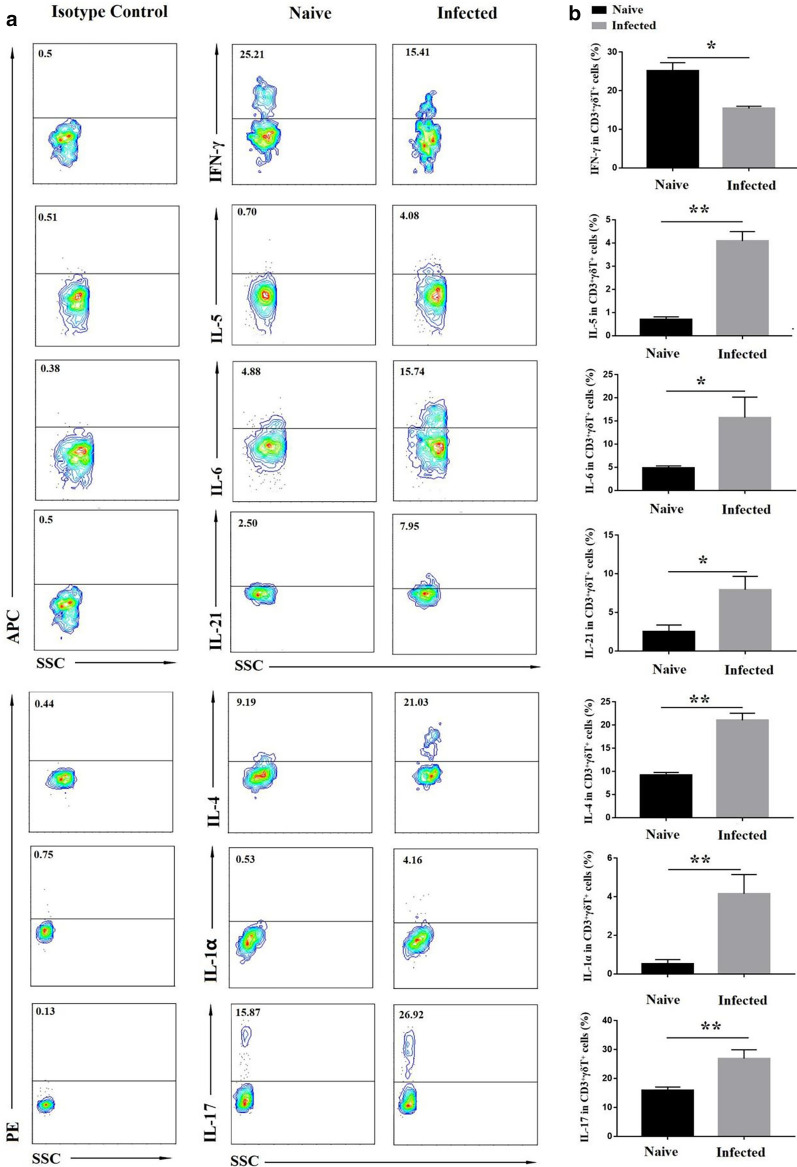
The expression of different cytokines from γδT cells. Pulmonary cells were stimulated with PMA and ionomycin. The ability of γδT cells to secrete cytokines (IFN-γ, IL-5, IL -6, IL -21, IL -4, IL -1, and IL -17) was detected. FMO controls for CD3, γδTCR and isotype controls for IFN-γ, IL-5, IL -6, IL -21, IL -4, IL -1, and IL -17 were included in the staining protocol. **a** One representative FCM analysis. **b** Comparison of the expression of different cytokines on γδT cells from the naive and infected groups. 5–7 samples were prepared for each group, and the experiments were repeated three times. *p < 0.05, **p < 0.01

To investigate the cytokine expression of γδT cells, pulmonary cells were stimulated with PMA and ionomycin, then stained for intracellular cytokines labelled with fluorescence. The staining strategy is shown in Additional file 1: Table S3. As shown in Fig. 3, the percentage of IFN-γ-expressing γδT cells from the infected mice was lower than that from the naive mice (*p* < 0.05), while the percentages of γδT cells that were expressing IL-5, IL -6, IL-21, IL-4, IL-1α, and IL-17 in the infected mice were higher than that in the naive mice (*p* < 0.05).

### Pathological changes of the lung in γδT KO mice

**Fig. 4:**
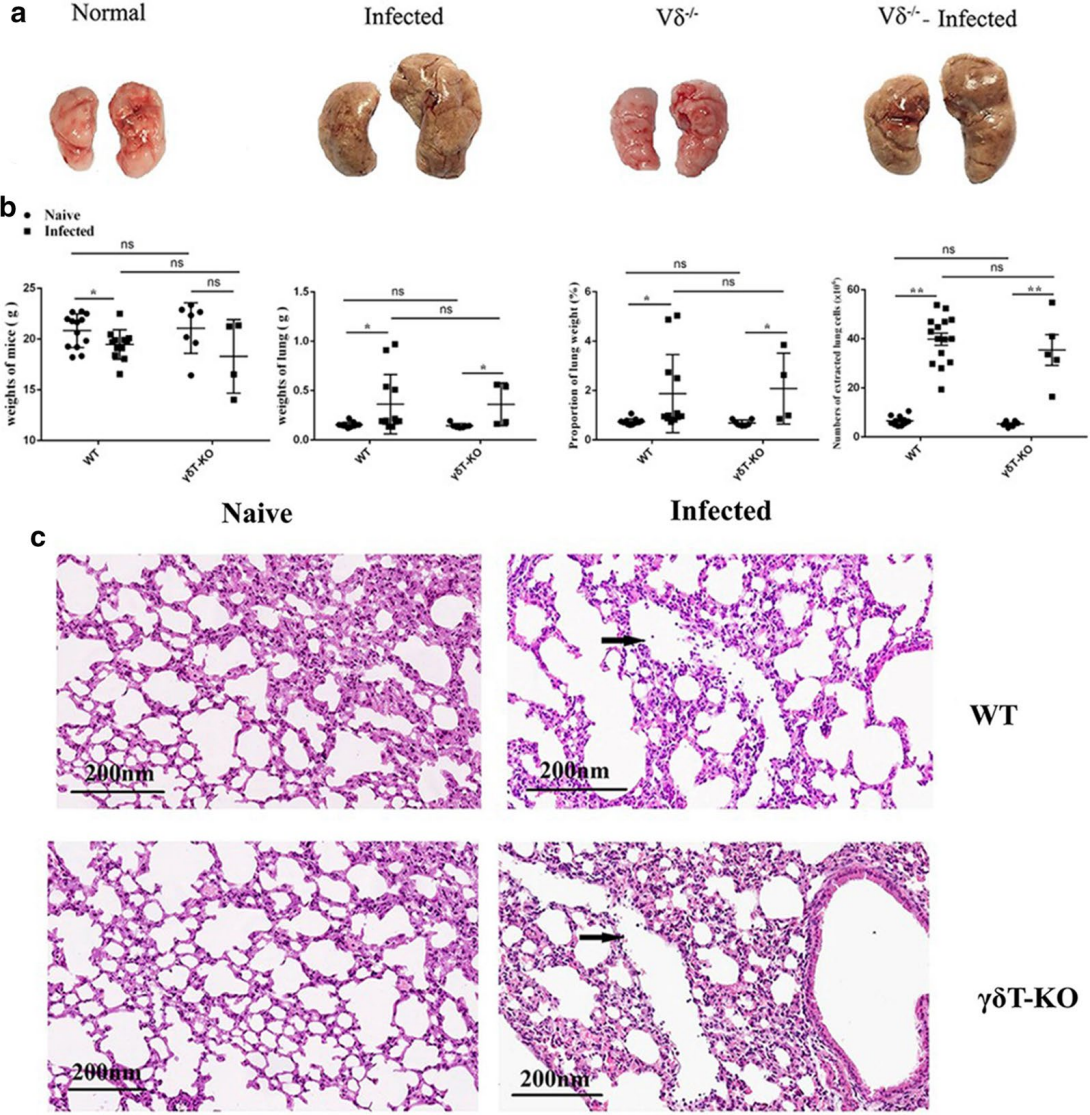
Pulmonary lesions in γδT KO mice infected with *Plasmodium. ***a** Comparison of the lung appearance in WT and γδT KO mice. Representative samples were shown for each group. **b** Comparison of the weight of the mice, the weight of the lung, the proportion of lung weight, and numbers of extracted lung cells from uninfected or infected WT and γδT KO mice. **c** HE staining of the lung tissue. The leukocyte infiltration is indicated by black arrows. Scale bar, 200 µm. *p < 0.05, **p < 0.01

To evaluate the role of γδT cells in *Plasmodium* infection, WT and γδT KO mice were injected with the same amount iRBCs. Mice were euthanized 11 days post-infection, and the lungs were removed. As shown in Fig. 4a, the size and colour of the lungs in the infected group is bigger and darker compared with the uninfected group, while, it was no obvious difference between the infected WT mice and infected γδT KO mice. The infected WT mice exhibited significant weight loss compared with uninfected WT mice (*p* < 0.05). The weight of the lung, the proportion of lung weight, and numbers of extracted lung cells from infected mice were higher than that from the uninfected mice (*p* < 0.05), there was no obvious difference between the infected WT and infected γδT KO mice (*p* > 0.05) (Fig. 4b). As shown in Fig. 4c with HE staining, the structure of the lung in the uninfected mice was clear with a uniform distribution of lung cells, but leukocyte infiltration and alveolar fusion were observed in the lung of the infected mice. However, the difference is not obvious between the infected WT and the infected γδT KO mice.

### The effect of γδT cells on T cells in the lung upon *Plasmodium* infection

**Fig. 5:**
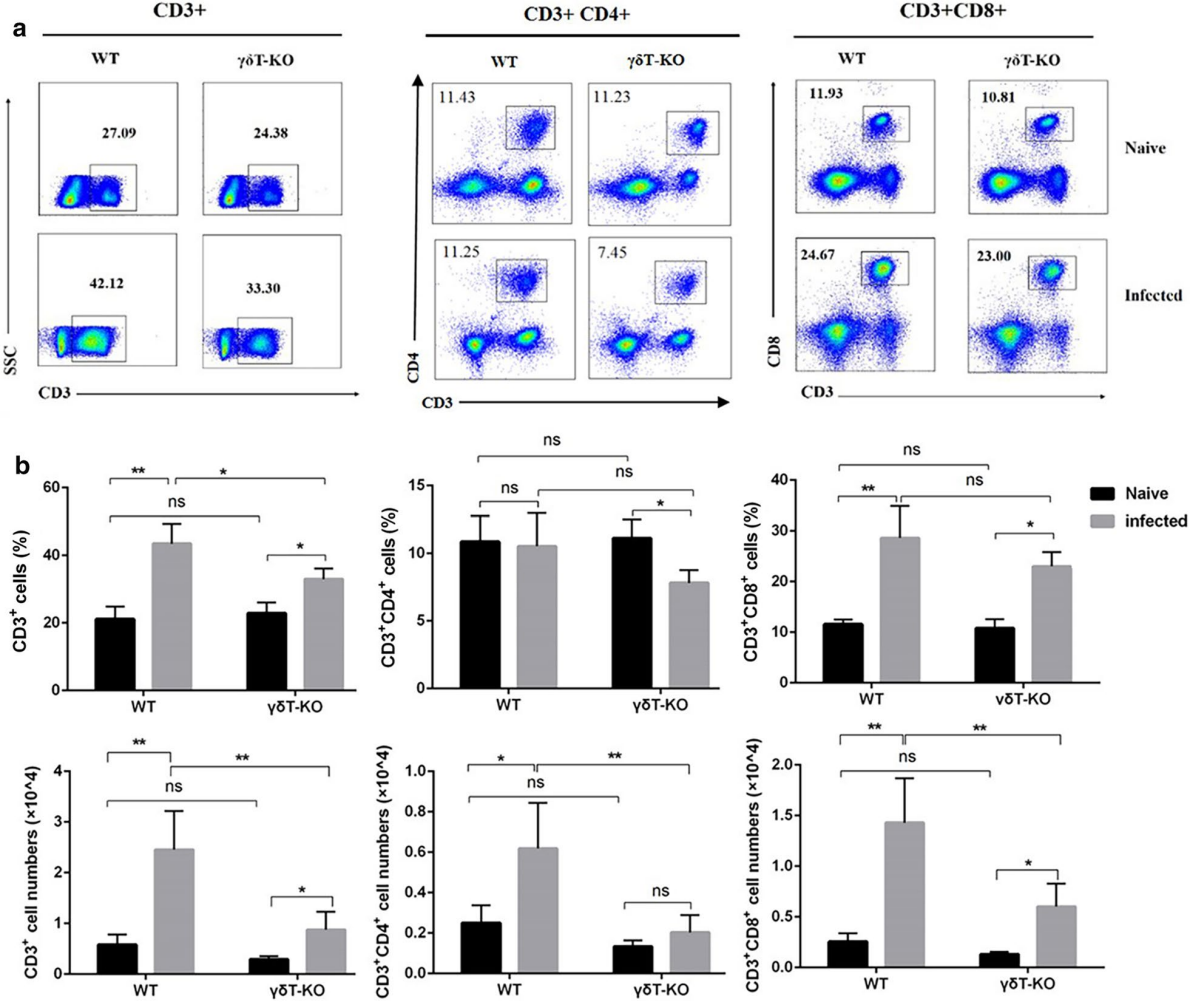
The content changes of T cells in the lung of γδT KO mice. Pulmonary cells from WT and γδT KO mice were stained with the different surface markers: CD3, CD4, and CD8. FMO controls for CD3, CD4, and CD8 were included in the staining protocol. **a** One representative FCM analysis for each comparison is shown. **b** Comparison of the percentage and the absolute number of CD3^+^, CD3^+^ CD4^+^, CD3^+^ CD8^+^ cells from the lung of WT and γδT KO mice. *p < 0.05, **p < 0.01

To elucidate the potential regulating role of γδT cells on T cells upon *Plasmodium* infection. The content, surface active molecular expression, and cytokine-producing ability of T cells from the lung of infected or uninfected WT and γδT KO mice were compared. As shown in Fig. 5, the single pulmonary cells were stained with different surface markers: CD3, CD4, and CD8. For gating CD3^+^ cells, CD3^+^ CD4^+^ cells, CD3^+^ CD8^+^ cells, FMO controls were used. The staining strategy is shown in Additional file 1: Table S4. The proportion and absolute numbers of CD3^+^ cells and CD3^+^ CD8^+^ cells from infected mice were higher than that from the uninfected mice (*p* < 0.05). Moreover, the proportion of CD3^+^ cells, and absolute numbers of CD3^+^ cells, CD3^+^ CD4^+^ cells, CD3^+^ CD8^+^ cells were decreased in the γδT KO infected mice compared with the WT infected mice (*p* < 0.05). These results indicated that γδT cells could promote the production of T cells upon *Plasmodium* infection.

**Fig. 6:**
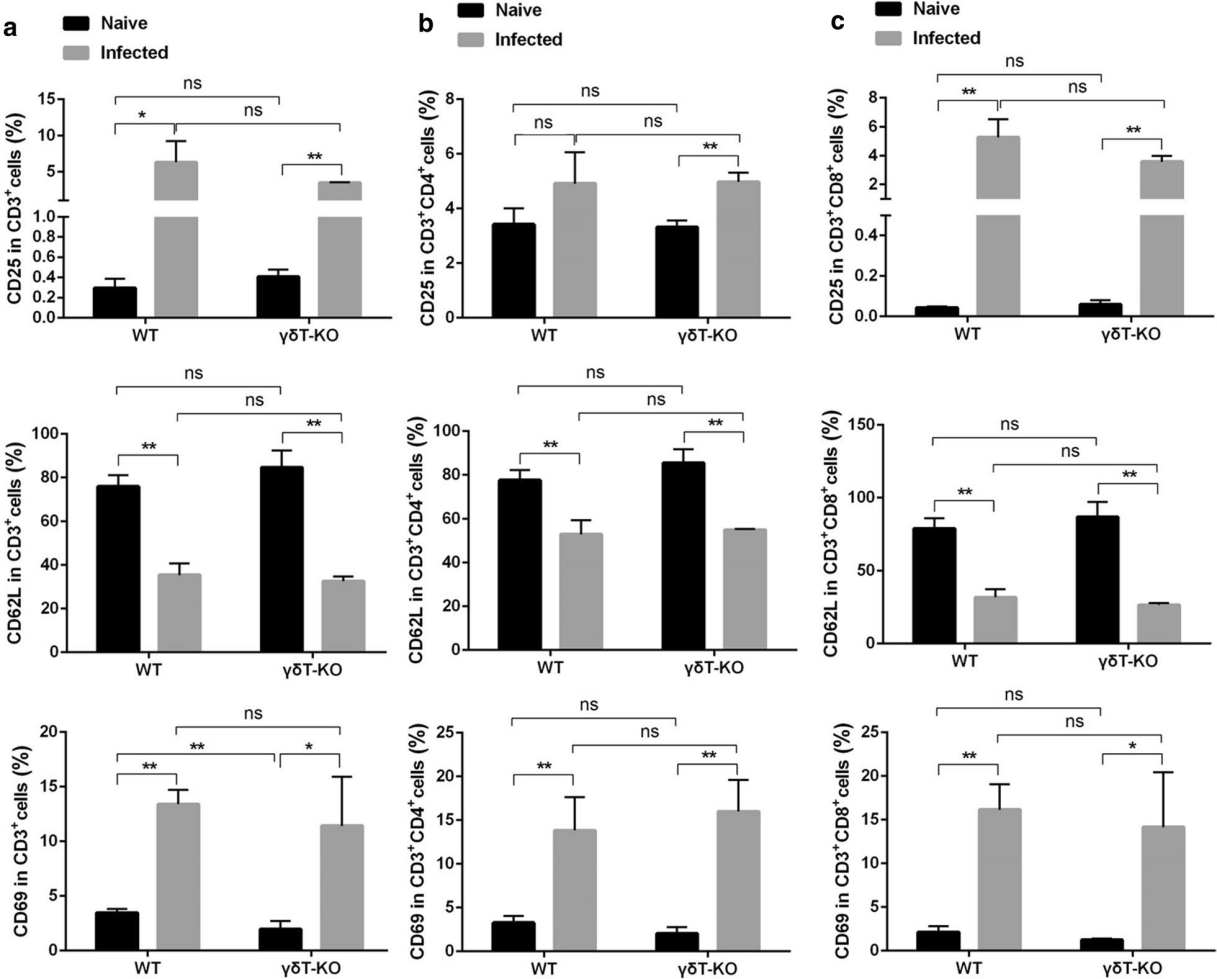
The expression of active surface markers on T cells. The surface active molecules expression of T cells from the lung of WT and γδT KO mice were calculated from FCM data and compared. FMO controls for CD3, CD4, and CD8 and isotype controls for CD69, CD25, and CD62L were included in the staining protocol. The average expression of CD69, CD25, and CD62L on CD3^**+**^ cells (**a**), CD3^**+**^ CD4^**+**^cells (**b**), and CD3^**+**^ CD8^**+**^cells (**c**). *p < 0.05, **p < 0.01

**Fig. 7:**
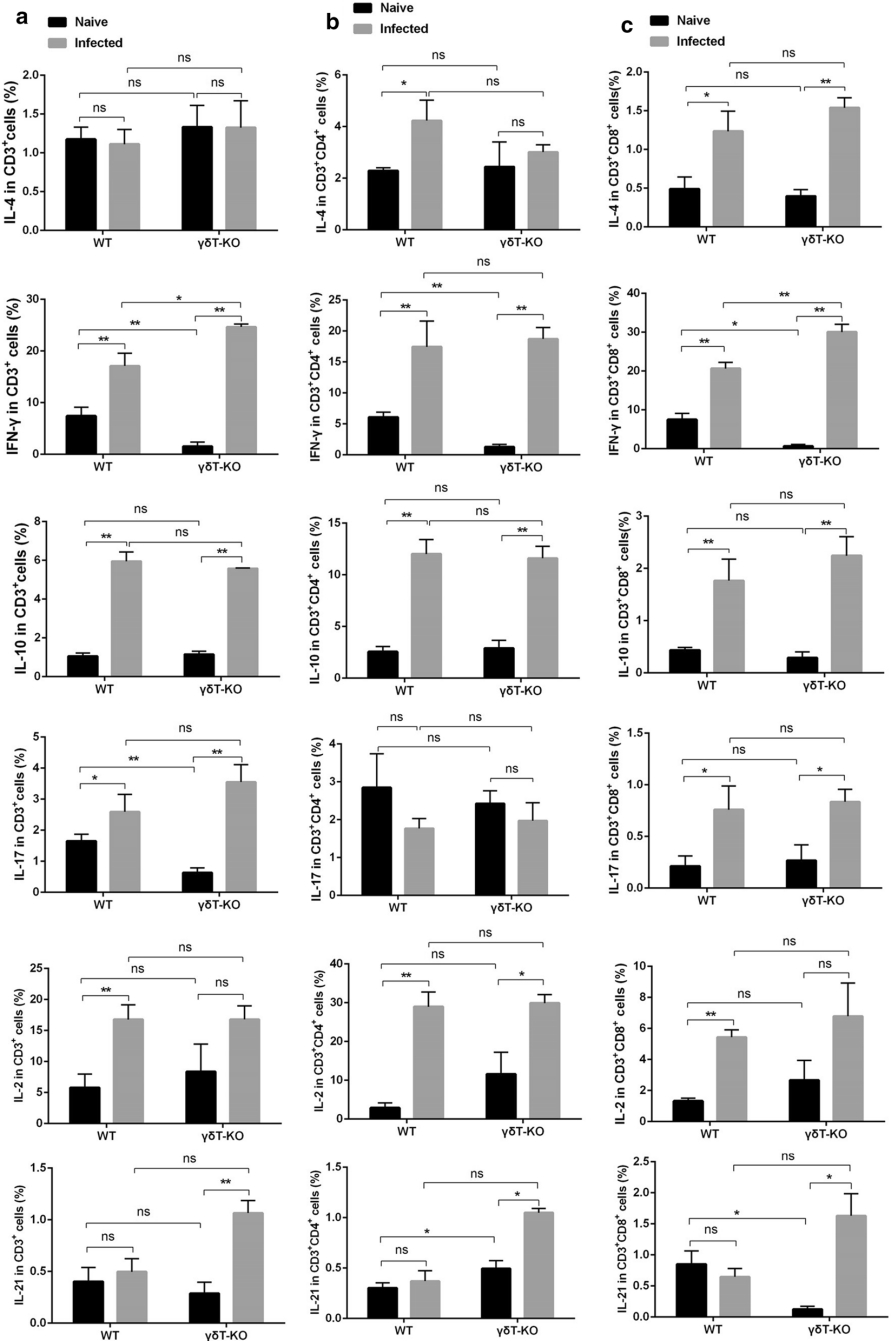
The cytokine-producing ability of CD3^+^, CD3^+^ CD4^+^, CD3^+^ CD8^+^ cells. The expression of IL-4, IFN-γ, IL-10, IL-17, IL-2, and IL-21 on T cells from the lung of WT and γδT KO mice were calculated from FCM data and compared. FMO controls for CD3, CD4, and CD8 and isotype controls for IL-4, IFN-γ, IL-10, IL-17, IL-2, and IL-21 were included in the staining protocol. The average expression of IL-4, IFN-γ, IL-10, IL-17, IL-2, and IL-21 on CD3 + cells (**a**), CD3^**+**^ CD4^**+**^cells (**b**), and CD3^**+**^ CD8^**+**^cells (**c**). *p < 0.05, **p < 0.01

Additionally, the phenotypic changes of T cells were investigated. The single pulmonary cells were stained with different antibodies labelled with fluorescence: CD3, CD4, CD8, CD69, CD62L, and CD25. The staining strategy is shown in Additional file 1: Table S5. As shown in Fig. 6a, more CD3^+^ T cells were expressing CD25 and CD69 (*p* < 0.05) and fewer CD3^+^ cells were expressing CD62L (*p* < 0.01) in infected mice when compared with the uninfected mice. This study demonstrated that mice lacking γδT cells and the corresponding wild-type strain do not differ in the percentages of CD25^+^ CD3^+^ T cells and CD62L^+^ CD3^+^ T cells (*p* > 0.05). The percentage of CD69^+^ CD3^+^ T cells decreased in uninfected γδT KO mice compared with uninfected WT mice (*p* < 0.01). For CD3^+^ CD4^+^ cells and CD3^+^ CD8^+^ cells (Fig. 6b and c), less of them were expressing CD62L (*p* < 0.01) and more of them were expressing CD69 (*p* < 0.01) in infected mice when compared with the uninfected mice. γδTCR knockout did not make a significant difference in the percentages of CD25^+^, CD62L^+^, and CD69^+^ CD3^+^ CD4^+^ T cells and CD3^+^ CD8^+^ T cells (*p* > 0.05). These results indicated that γδTCR knockout did not make a significant difference in the surface molecular expression of T cells. Furthermore, the cytokine-producing ability of T cells from the lung of infected or uninfected WT and γδT KO mice were compared. IL-4, IFN-γ, IL-10, IL-17, IL-2 and IL-21 from CD3^+^, CD3^+^ CD4^+^ and CD3^+^ CD8^+^ cells were detected. The staining strategy is shown in Additional file 1: Table S6. As shown in Fig. 7a, more CD3^+^ T cells were expressing IFN-γ, IL-10, and IL-17 in the infected mice when compared with the uninfected mice (*p* < 0.05). The percentage of IFN-γ^+^ T cells decreased in uninfected γδT KO mice compared with uninfected WT mice (*p* < 0.01) (Fig. 7a–c). It implied that the deficiency of γδT cells has some effects on cytokine-producing abilities of T cells in the absence of malaria infection. However, although the difference is statistically significant, it is very small which could not have a biological impact. Moreover, the percentage of IFN-γ- expressing CD3^+^ and CD3^+^ CD8^+^ cells from infected γδT KO mice was higher than from the infected WT mice (*p* < 0.05) (Fig. 7). These indicated that γδT cells could suppress T cells to produce IFN-γ upon *Plasmodium* infection. When γδT cells were knockout, more T cells could produce IFN-γ upon *Plasmodium* infection.

### The effect of γδT cells on B cells in the lung upon ***Plasmodium*** infection

**Fig. 8:**
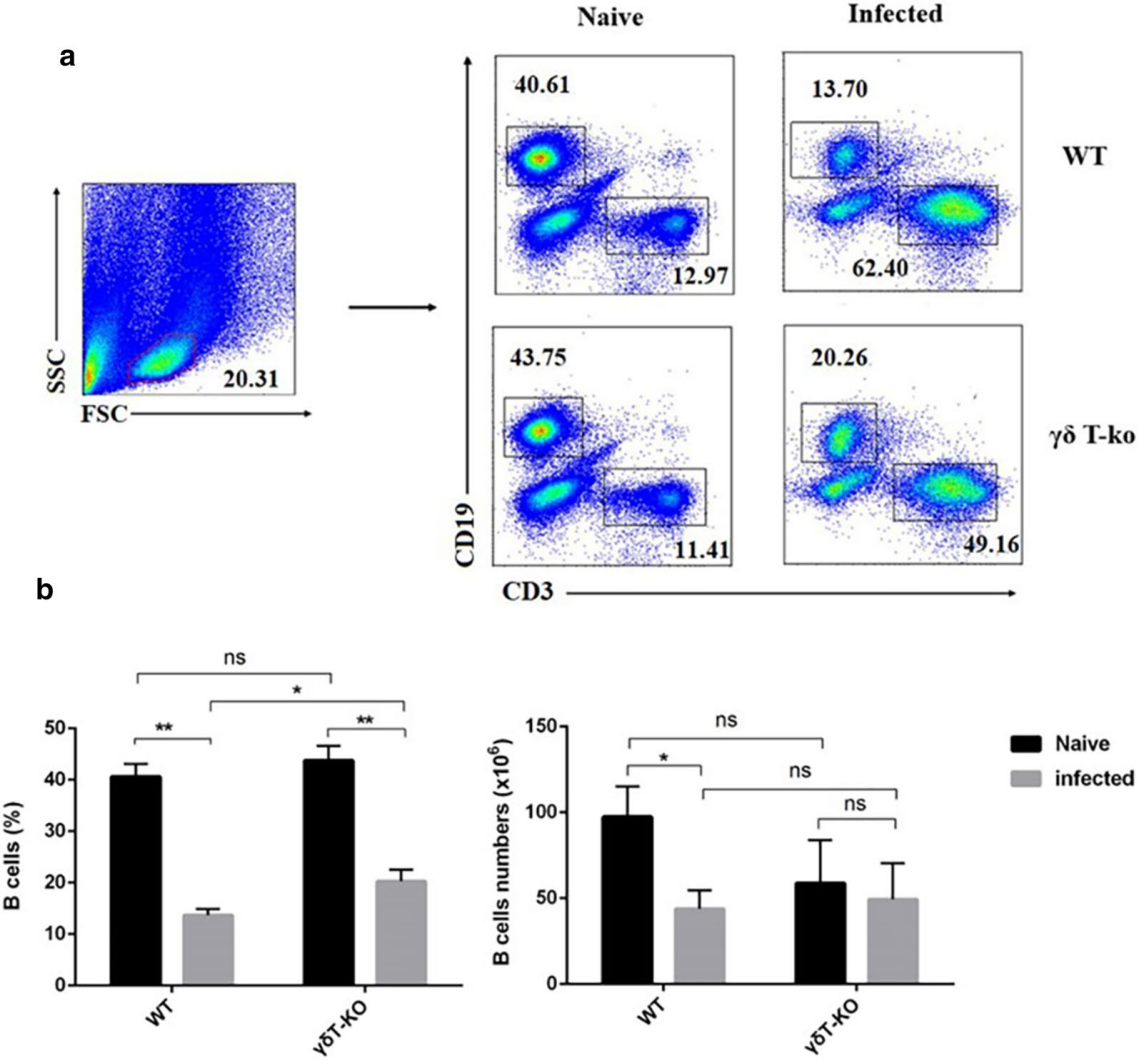
The content changes of B cells in the lung of γδT KO mice. Pulmonary cells from WT and γδT KO mice were stained with fluorescent mAbs against mice CD19, CD13. FMO controls for CD19 and CD3 were included in the staining protocol. **a** One representative FCM analysis for the comparison is shown. **b** Comparison of the percentage and absolute numbers of CD3^−^ CD19^+^ cells from the lung of WT and γδT KO mice. *p < 0.05, **p < 0.01

To further explore the potential modulating role of γδT cells on B cells upon *Plasmodium* infection. The content, surface active molecular expression of B cells from the lungs were compared. The single pulmonary cells were stained with fluorescence-labelled surface markers: CD3, CD19, CD69, ICOS, and CD80. The staining strategy is shown in Additional file 1: Table S7–8. As shown in Fig. 8, the proportion of CD19^+^ cells from the infected mice decreased compared with uninfected mice (*p* < 0.05), while it was increased in the γδT KO infected mice compared with the WT infected mice (*p* < 0.05). The absolute numbers of CD19^+^ cells were no obvious change in the infected WT and the infected γδT KO mice (*p* > 0.05). These results indicated that the deletion of γδT did not affect the proliferation of B cells.

**Fig. 9:**
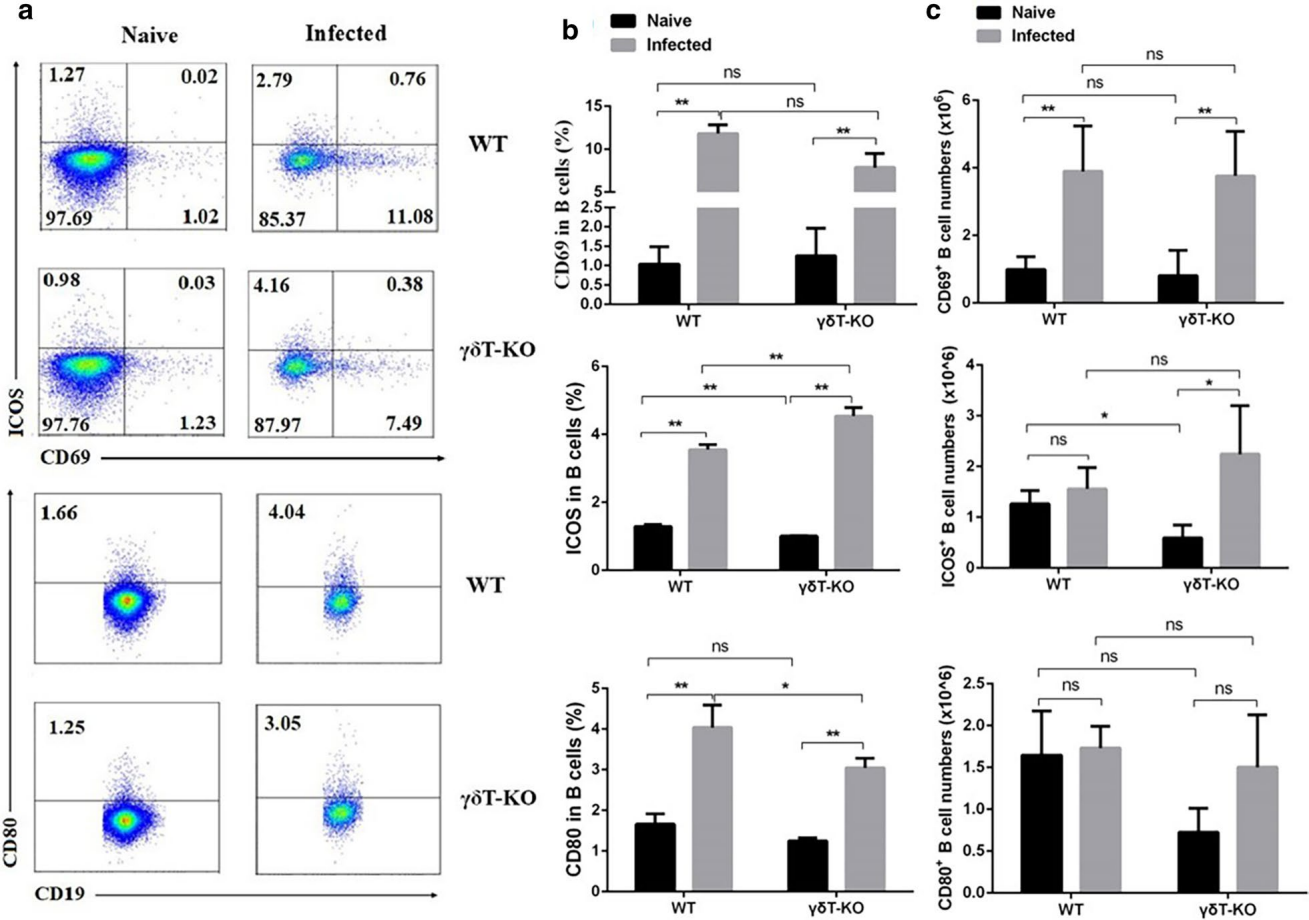
The expression of active surface markers on B cells. The surface molecules expression of B cells was calculated from FCM data and compared. FMO controls for CD19 and CD3 and isotype controls for CD69, ICOS, and CD80 were included in the staining protocol. **a** One representative FCM analysis for each comparison is shown. **b** Comparison of the percentage of CD69^**+**^, ICOS^**+**^, and CD80^+^ B cells from the lung of WT and γδT KO mice. *p < 0.05, **p < 0.01

As shown in Fig. 9, the percentages of CD69, ICOS, or CD80-expressing B cells increased in infected mice during the course of infection (*p* < 0.01). It showed that a higher percentage of ICOS-expressing B cells and a lower percentage of CD80-expressing B cells from infected γδT KO mice compared with infected WT mice (*p* < 0.05). However, there was no obvious difference for the absolute numbers of CD69^+^, ICOS^+^, and CD80^+^ B cells between infected WT and infected γδT KO mice (*p* > 0.05). Taken together, these results indicated that the deletion of γδT did not significantly affect B cells’ immune response upon *Plasmodium* infection.

## Discussion

γδT cells comprise a small population of T cells (3–5%) [32]. In this study, the characteristics of γδT cells from the lungs of *P. yoelii* infected C57BL/6 mice were explored. The percentage and the absolute number of γδT cells were significantly increased (Fig. 1) in the lungs of *P. yoelii* infected C57Bl/6 mice at 11 days post-infection. Similarly, Mamedov et al. also reported γδT cells are expanded in the lungs of *Plasmodium chabaudi* infected C57Bl/6 mice at 16 days post-infection when parasite recrudescence reached a peak in the mice whose γδT cells were silenced [22]. These results indicated that γδT cells accumulate in the lung and may play a role in the process of host anti-*Plasmodium* infection.

To study the potential role of CD3^+^ γδTCR^+^ cells after infection, the phenotype of γδT cells was examined. CD127, CD34 and CD62L are T cell activation-associated molecules [33–35]. CD34 serves as a ligand for CD62L, CD34 and CD62L primarily regulates the proliferation and migration of leukocytes to inflammatory sites and lymph nodes [33, 34]. The percentages of CD62L^+^, CD127^+^, and CD34^+^ γδT cells decreased significantly in the infected group (*p* < 0.05). PD-1 acts as an inhibitory receptor, which could reduce T cell receptor (TCR) induced cell proliferation, cytokine production, and cytolytic activity [36]. MHC II, CD80, and CD11b are the surface markers of antigen-presenting cells, which could accelerate T-cell activation [37, 38]. More γδT cells were expressing CD80, CD11b and PD-1 post-infection (*p* < 0.05). While the percentages of MHC II^+^ and PD-L1^+^ γδT cells did not significantly change post-infection (*p* > 0.05). These results further confirm that γδT cells could regulate the inflammation response in the lung of *P. yoelii* infected mice, and γδT cells may be beneficial for antigen presentation in the lung of infected mice.

It is reported that γδT cells could secrete numerous cytokines to mediate the immune response [19]. In this study, the results showed more γδT cells secrete Th2 cytokines (IL-4, IL-5), IL -6, IL -21, IL -1α, IL -17, and fewer γδT cells secrete IFN-γ in response to *Plasmodium* infection. It implied that the Th2 immune response is promoted by increased IL-4 and IL-5 secreted from γδT cells. As a pro-inflammatory cytokine, IL-17 extensively participated in host antimicrobial immunity [39–41]. It is commonly accepted that IL-17 is predominately produced by γδT cells upon *Mycobacterium tuberculosis* infection [42]. IL-21 is a pleiotropic cytokine, which is related to autoimmune diseases, allergies, and inflammatory diseases. It can enhance the body’s adaptive immune response and innate immune response [43]. IL-1α and IL-6 are required for Th17 lymphocyte differentiation upon host infected with *Paracoccidioides brasiliensis* [44]. In this study, the percentage of IL-6, IL-21, IL-1α, IL-17 γδT cells increased significantly after infection (*p* < 0.05). These data indicated that γδT cells could promote host immune response in anti-*P. yoelii* infection.

To explore the role of γδT cells in *Plasmodium* infection-induced lung injury, γδT KO mice were infected with *P. yoelii*. γδT cells, one functional group of cells, has some effects on phenotypes and cytokine-producing abilities of T and B cells. For example, the percentages of CD69^+^ CD3^+^ T cells, IFN-γ- expressing T cells, IL-17 expressing CD3^+^ T cells, ICOS^+^ B cells decreased in γδT KO mice in the absence of malaria infection (*p* < 0.05) (Figs. 6, 7, 8 and 9). Even so, the γδT KO mice are still the best model to study the role of γδT cells. There was no obvious difference in the lung between the WT and γδT KO mice in either the uninfected or the infected group (Fig. 4). One potential reason for this phenomenon may be the lower percentage of γδT cells in the lung. Although it plays a certain role in anti-*P. yoelii* infection, the deletion of γδ TCR is not enough to alter the pathological damage of the lung. T cell response was studied in the *P. yoelii-*infected WT and γδT KO mice. T cell-mediated immunity is the key for the host to defense against malaria parasite infection [45]. Parasite-specific CD8^+^ T cells participate in the process of malaria-associated ALI and ARDS by promoting pulmonary vascular leakage and pulmonary oedema [15, 16]. There was no significant difference for the proportion and absolute numbers of T cells between uninfected WT mice and the uninfected γδT KO mice (*p* > 0.05). However, the proportion of CD3^+^ cells and the absolute numbers of CD3^+^ cells, CD3^+^ CD4^+^ cells, CD3^+^ CD8^+^ cells were decreased in γδT KO infected mice compared with the WT infected mice (*p* < 0.05). These results indicated that γδT cells could promote the recruitment of T cells upon *P. yoelii* infection. The possible reason maybe the secretion of chemokines by γδT cells, like M-CSF. It is commonly accepted that the chemokine system plays critical role in the recruitment of lymphocytes [22, 46].

The deficiency in γδT cells did not make a significant difference on the surface molecular expression of T cells for the infected mice (*p* > 0.05), suggesting that γδT cells were not associated with the activation of T cells. IFN-γ is the central molecule in mediating host protective immune responses against malaria parasites [47]. The percentage of IFN-γ- expressing CD3^+^ and CD3^+^ CD8^+^ cells increased in γδT KO infected mice compared with the WT infected mice (*p* < 0.05). These results indicated that γδT cells could suppress the production of IFN-γ in CD3^+^ and CD3^+^ CD8 ^+^ cells upon *P. yoelii* infection. Taken together, γδT cells played double effects on T cells, especially CD3^+^ CD8 ^+^ cells, mediated anti-malarial response in the lung.

Additionally, B cell response was also investigated, the absolute number of B cells was not affected by γδ TCR knockout. The B cells expressed ICOS could induce regulatory T cells [48]. Many types of antigen-presenting cells can express CD80 [49] and the expression of CD80 in B cells plays a critical role in regulating B-T interactions in both early and late germinal center responses [50]. Although the percentages of ICOS and CD80-expressing B cells differed in infected γδT KO mice compared with the infected WT mice (*p* < 0.05). The absolute numbers of ICOS^+^, CD69^+^, and CD80^+^ B cells were not significantly changed in the infected γδT KO mice compared with the infected WT mice (*p* > 0.05). It is suggested that γδT cells may not contribute to the proliferation and phenotype changes of B cells upon *P. yoelii* infection.

## Conclusions

This study explored the phenotypic and functional characteristics of γδT cells in the lung of *P. yoelii*-infected mice, and found that *Plasmodium* infection can induce significant changes in the content, phenotype, and function of the lung γδT cells, γδT cells contribute to T cell immune response in the lungs of mice infected with *Plasmodium*. Although there are differences in tissue distributions and TCR development between mice and humans, the primary theme for γδ T cells in protecting against disease and tissue damage is basically conserved [51]. This study is beneficial for understanding the roles of γδT cells in malaria patients.

## Supplementary Information

**Additional file 1.** Additional Tables.

## Data Availability

The datasets used and/or analysed during the current study are available from the corresponding author on reasonable request.

## Abbreviations

WT: Wild-type
TCR: T cell receptor
iRBCs: Infected red blood cells
HE: Haematoxylin-eosin
HBSS: Hank’s balanced salt solution
γδT cells: Gamma delta T cells
ALI: Acute lung injury
ARDS: Acute respiratory distress syndrome
γδT KO: γδTCR knockout
FMO: Fluorescence minus one
PMA: Phorbol 12-myristate 13-acetate
