# Supplementary material for: Characterization of γδT cells in lung of Plasmodium yoelii-infected C57BL/6 mice
Source: Malar J. 2021 Feb 15;20:89. doi: 10.1186/s12936-021-03619-z (PMC7885449; doi:10.1186/s12936-021-03619-z)
Supplement: Supplementary file 1 — Additional file 1. Additional Tables. [file 12936_2021_3619_MOESM1_ESM.docx]

**Table S1. The staining strategy for figure 1.**

| **Tubes** | **APC-cy7** | **FITC** |
| --- | --- | --- |
| FMO Ctrl 1 | CD3 | - |
| FMO Ctrl 2 | - | γδ TCR |
| Test 1 | CD3 | γδ TCR |

Note: - blank

**Table S2. The staining strategy for figure 2.**

| **Tubes** | **APC-cy7** | **FITC** | **PE-cy7** | **APC** | **PE** | **Brilliant Violet 421** |
| --- | --- | --- | --- | --- | --- | --- |
| FMO Ctrl 1 | CD3 | - | - | - | - | - |
| FMO Ctrl 2 | - | γδ TCR | - | - | - | - |
| Isotype Ctrl 1 | - | - | Isotype Ctrl | - | - | - |
| Isotype Ctrl 2 | - | - | - | Isotype Ctrl | - | - |
| Isotype Ctrl 3 | - | - | - | - | Isotype Ctrl | - |
| Isotype Ctrl 4 | - | - | - | - | - | Isotype Ctrl |
| Test 1 | CD3 | γδ TCR | - | - | - | - |
| Test 2 | CD3 | γδ TCR | CD11b | CD62L | CD80 | - |
| Test 3 | CD3 | γδ TCR | - | CD34 | CD127 | - |
| Test 4 | CD3 | γδ TCR | - | - | PD-1 | PD-L1 |

Note: - blank

**Table S3. The staining strategy for figure 3.**

| **Tubes** | **APC-cy7-** | **FITC** | **APC** | **PE** |
| --- | --- | --- | --- | --- |
| FMO Ctrl 1 | CD3 | - | - | - |
| FMO Ctrl 2 | - | γδ TCR | - | - |
| Isotype Ctrl 1 | - | - | Isotype Ctrl | - |
| Isotype Ctrl 2 | - | - | - | Isotype Ctrl |
| Test 1 | CD3 | γδ TCR | - | - |
| Test 2 | CD3 | γδ TCR | IFN-γ | IL-4 |
| Test 3 | CD3 | γδ TCR | IL-21 | IL-17 |
| Test 4 | CD3 | γδ TCR | IL-6 | IL-1α |
| Test 5 | CD3 | γδ TCR | IL-5 | - |

Note: - blank

**Table S4. The staining strategy for figure 5.**

| **Tubes** | **APC-cy7** | **FITC** | **Percp-cy5.5** |
| --- | --- | --- | --- |
| FMO Ctrl 1 | CD8 | - | - |
| FMO Ctrl 2 | - | CD3 | - |
| FMO Ctrl 3 | - | - | CD4 |
| Test 1 | CD8 | CD3 | CD4 |

Note: - blank

**Table S5. The staining strategy for figure 6.**

| **Tubes** | **APC-cy7** | **FITC** | **Percp-cy5.5** | **Brilliant Violet 421** | **PE** | **APC** |
| --- | --- | --- | --- | --- | --- | --- |
| FMO Ctrl 1 | CD8 | - | - | - | - | - |
| FMO Ctrl 2 | - | CD3 | - | - | - | - |
| FMO Ctrl 3 | - | - | CD4 | - | - | - |
| Isotype Ctrl 1 | - | - | - | Isotype Ctrl | - | - |
| Isotype Ctrl 2 | - | - | - | - | Isotype Ctrl | - |
| Isotype Ctrl 3 | - | - | - | - | - | Isotype Ctrl |
| Test 1 | CD8 | CD3 | CD4 | - | - | - |
| Test 2 | CD8 | CD3 | CD4 | CD69 | CD25 | CD62L |

Note: - blank

**Table S6. The staining strategy for figure 7.**

| **Tubes** | **APC-cy7** | **FITC** | **Percp-cy5.5** | **PE** | **APC** |
| --- | --- | --- | --- | --- | --- |
| FMO Ctrl 1 | CD8 | - | - | - | - |
| FMO Ctrl 2 | - | CD3 | - | - | - |
| FMO Ctrl 3 | - | - | CD4 | - | - |
| Isotype Ctrl 1 | - | - | - | Isotype Ctrl | - |
| Isotype Ctrl 2 | - | - | - | - | Isotype Ctrl |
| Test 1 | CD8 | CD3 | CD4 | - | - |
| Test 2 | CD8 | CD3 | CD4 | IL-4 | IFN-γ |
| Test 3 | CD8 | CD3 | CD4 | IL-10 | IL-17 |
| Test 4 | CD8 | CD3 | CD4 | IL-2 | IL-21 |

Note: - blank

**Table S7. The staining strategy for figure 8.**

| **Tubes** | **FITC** | **Percp-cy5.5** |
| --- | --- | --- |
| FMO Ctrl 1 | CD3 | - |
| FMO Ctrl 2 | - | CD19 |
| Test 1 | CD3 | CD19 |

Note: - blank

**Table S8. The staining strategy for figure 9.**

| **Tubes** | **FITC** | **Percp-cy5.5** | **Brilliant Violet 421** | **PE** | **PE-cy7** |
| --- | --- | --- | --- | --- | --- |
| FMO Ctrl 1 | CD3 | - | - | - | - |
| FMO Ctrl 2 | - | CD19 | - | - | - |
| Isotype Ctrl 1 | - | - | Isotype Ctrl | - | - |
| Isotype Ctrl 2 | - | - | - | Isotype Ctrl | - |
| Isotype Ctrl 3 | - | - | - | - | Isotype Ctrl |
| Test 1 | CD3 | CD19 | - | - | - |
| Test 2 | CD3 | CD19 | CD69 | CD80 | ICOS |

Note: - blank
